# Anaemia in the Hospitalized Elderly in Tanzania: Prevalence, Severity, and Micronutrient Deficiency Status

**DOI:** 10.1155/2021/9523836

**Published:** 2021-02-26

**Authors:** Clara Chamba, Ahlam Nasser, William F. Mawalla, Upendo Masamu, Neema Budodi Lubuva, Erius Tebuka, Pius Magesa

**Affiliations:** ^1^Department of Haematology and Blood Transfusion, Muhimbili University of Health and Allied Sciences (MUHAS), Dar-es-salam, Tanzania; ^2^Department of Internal Medicine, Muhimbili National Hospital (MNH), Dar-es-salaam, Tanzania; ^3^Department of Pathology, Catholic University of Health and Allied Sciences (CUHAS), Mwanza, Tanzania

## Abstract

**Introduction:**

Anaemia is a common problem in sub-Saharan Africa. While most literature has focused on children, women of childbearing age, and pregnant women, data for the elderly population are relatively scarce. Anaemia exhorts negative consequences to functional ability of elderly patients, both physically and cognitively. The purpose of this study was to determine the prevalence of anaemia, severity, and micronutrient deficiency status in the elderly hospitalized patients in Tanzania.

**Methods:**

A total of 156 hospitalized adults aged 60 years and above were enrolled in this study. A structured questionnaire was used to capture sociodemographic and clinical characteristics. Blood samples were collected, and a complete blood count, serum cobalamin, serum ferritin, and serum folate levels were measured to assess anaemia and micronutrient deficiency status in all participants who had anaemia.

**Results:**

The prevalence of anaemia was 79.5% (124/156) with severe anaemia in 33.9% (42/124) of participants, moderate anaemia in 42.7% (53/124) of participants, and 23.4% (29/124) of all participants had mild anaemia. Micronutrient deficiency was found in 14.5% (18/124) of all participants with anaemia. Combined deficiency (either iron and vitamin B12 deficiency or iron and folate deficiency) was the most common micronutrient deficiency anaemia with a frequency of 33.3% (6/18), followed by isolated iron and folate deficiencies at equal frequency of 27.8% (5/18) and vitamin B12 deficiency at 11.1% (2/18).

**Conclusion:**

The prevalence of anaemia in the hospitalized elderly population is high warranting public health attention and mostly present in moderate and severe forms. Micro-nutrient deficiency anaemia is common in this age group and is mostly due to combined micronutrient deficiency.

## 1. Introduction

Anaemia is a condition that presents with a decrease in the population of red blood cells in the body [1]. The widely used method for establishing anaemia is through measurement of hemoglobin concentration in the blood [1, 2]. The World Health Organization (WHO) defines anaemia as hemoglobin of less than 13 g/dl in men and less than 12 g/dl in women [2, 3]. It further classifies the anaemia severity into mild, moderate, and severe based on the hemoglobin levels [2]. Whilst this definition is being applied for all populations, different studies argue that the data used excluded individuals above 65 years of age [4–6]. Nevertheless, in adults aged 60 years and above, anaemia has notable adverse consequences of impaired functionality, cognition, increased hospital admissions, and increased morbidity and mortality [7, 8]. The WHO estimates that roughly 24% of older adults (over 60 years of age) globally have anaemia [3]. In developed countries, the prevalence of anaemia in the elderly aged 60 years and above ranges between 3% and 63% with a higher prevalence found in those who are hospitalized or in nursing homes [5, 9–11]. Prevalence of anaemia in the elderly population is higher in developing countries, ranging between 20.6% and 49.5% in community based studies [12–16] and those in nursing homes having a prevalence as high as 68.7 [17, 18]. A community survey done in Uganda in 2013 revealed 20% of adults aged over 50 years were anaemic [19]. There is generally a paucity of data for the prevalence of anaemia in hospitalized elderly from developing countries, particularly in Africa. Anaemia has been extensively studied in children, women of reproductive age, and pregnant women in Tanzania and found to be high and mostly attributable to nutritional causes [20–22]. However, to the best of our knowledge, there are no studies that have been done on anaemia in the elderly population in Tanzania.

## 2. Materials and Methods

### 2.1. Study Design

This was a hospital-based descriptive cross-sectional study which recruited patients aged 60 years and above admitted at Muhimbili National Hospital (MNH), between September 2015 and February 2016. MNH is the main national referral hospital receiving patients from all over Tanzania.

### 2.2. Participant Enrollment and Data Collection

**Figure 1:**
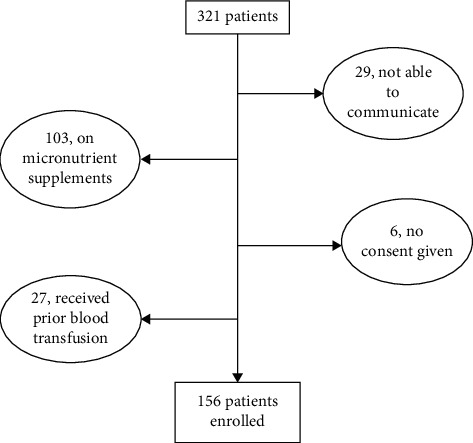
Enrollment Flow chart.

Participants were enrolled to the study if they were 60 years or older, admitted at MNH, and consented to participate in the study. During the study period, 321 patients over the age of 60 were admitted. Of these, only 156 were eligible for inclusion in the study. Participants were excluded from the study if (1) they had received a blood transfusion seven days prior to their admission (27 participants); (2) they were on treatment for nutritional causes of anaemia such as oral iron, folate, or vitamin B_12_ (103 participants); or (3) they were not able to communicate (29 participants). Six participants did not give consent to participate in the study (Figure 1). The study was ethically approved by the Muhimbili University of Health and Allied Sciences (MUHAS) Research Ethics Committee, and written informed consent was obtained from all participants prior to enrollment. A structured questionnaire was filled for each participant, recording sociodemographics characteristics and clinical parameters.

### 2.3. Sample Collection and Laboratory Methods

Ten milliliters of venous blood was drawn for laboratory tests (haematology and biochemistry) from each participant within 24 hours of admission. Blood was collected into sterile vacutainers for haematological tests (EDTA anticoagulant) and biochemical tests (vacutainers without additives). Haematological tests were run at the Central Pathology Laboratory at MNH. Full blood counts were run on a 3700 CELL DYN machine. Serum vitamin B12 and serum ferritin tests were done on STAT FAX 303 ELISA SYSTEM, and serum folate levels were measured on ARCHITECT PLUSCI 4100 machine. The quantitative determination of serum ferritin was done using the DRG Ferritin ELISA assay (EIA 4292).

### 2.4. Definition of Key Terms

Anaemia was defined as hemoglobin levels of less than 13.0 g/dl in men and less than 12.0 g/dl in women, based on the WHO definition. Severity of anaemia was categorized based on the WHO classification, whereby mild anaemia was denoted by hemoglobin levels of 11 g/dl–11.9 g/dl in women and hemoglobin 11–12.9 g/dl in men. Moderate anaemia is denoted if hemoglobin levels were between 8 and 10.9 g/dl in both men and women and severe anaemia if hemoglobin levels were less than 8 g/dl in both men and women [3].

Iron deficiency anaemia was denoted by hemoglobin levels <12 g/dl in females and <13 g/dl in males accompanied with a serum ferritin of less than 30 *μ*g/ml [23].

Vitamin B12 deficiency anaemia was considered in patients with hemoglobin levels <12 g/dl for women and <13 g/dl for men accompanied with a serum vitamin B12 of less than 148 pmol/L [24, 25].

Folate deficiency anaemia was defined as hemoglobin levels <12 g/dl in women and <13 g/dl in men accompanied with a serum folate <3 ng/ml [25].

Nutritional deficiency anaemia was considered in patients with hemoglobin levels <12 g/dl in women and <13 g/dl in males accompanied with either iron deficiency and folate deficiency or vitamin B12 deficiency [3].

### 2.5. Data Management and Statistical Analysis

Collected data were screened for quality, and coding was done prior to entering into R-studio statistical programs, which was used for analysis. Data analysis included calculation of means and standard deviations for numerical data which was normally distributed. Medians and interquartile ranges were computed for data which was not normally distributed. Categorical data were summarized by frequencies and proportions. Hypothesis testing was further undertaken using the Student's *t* test and chi-squared test for numerical and categorical variables, respectively. A *p* value <0.05 was considered statistically significant.

## 3. Results

### 3.1. Study Participants

The study enrolled 156 participants, of whom 95 (60.9%) were males. The median age was 66 years, with the oldest participant being 90 years old. More than two thirds were married, and half of them had no formal education. Overweight (BMI between 25.0 and <30) and obesity (BMI ≥30) were recorded in 44.8% of all participants, majority being males (71.4%) (Table 1).

### 3.2. Prevalence of Anaemia

**Figure 2:**
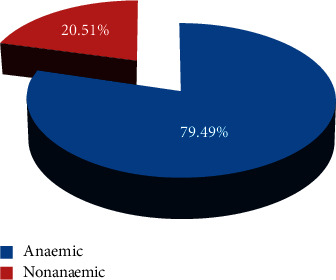
Prevalence of anaemia.

The overall prevalence of anaemia was 79.5% (95% CI 72.5–85.1%) (Figure 2). There was no significant difference in proportion of males with anaemia and females with anaemia, 80% (95% CI = 70.9–86.8%) versus 78.7% (95% CI = 66.9–87.1%), respectively, (*P* value = 1). There was no evidence of a trend in proportion of anaemia between males and females within different age groups (<70 years, 70–79 years, and 80+ years).

### 3.3. Severity of Anaemia

**Figure 3:**
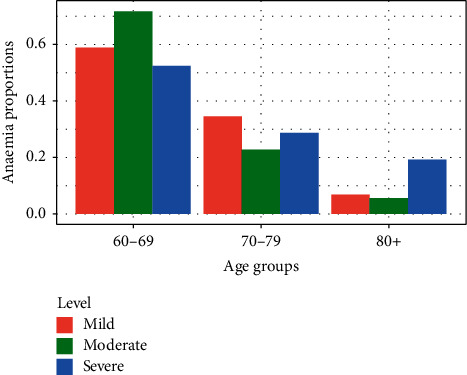
Severity of anaemia with age groups.

Moderate anaemia was found in 53 (42.7%) anaemic participants. It was followed by severe anaemia in 42 (33.9%) and mild anaemia in 29 (23.4%) anaemic participants. Moderate anaemia was the most common type of anaemia among participants aged 60–69 years. For participants who were older than 80 years, severe anaemia was more common (Figure 3).

### 3.4. Nutritional Deficiency Anaemia

**Figure 4:**
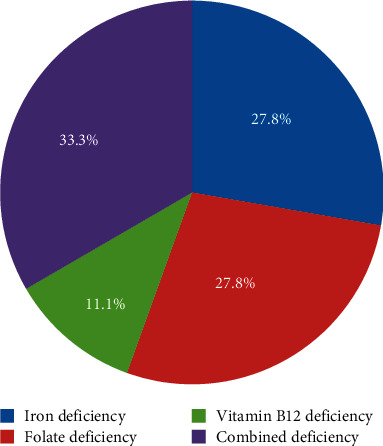
Proportion of nutritional deficiency anaemia, *N* = 18.

Among the participants with anaemia, a total of 18 (14.5%) had nutritional deficiency. Of those with nutritional deficiency, majority (6/18 (33.3%)) had combined deficiency (either iron and vitamin B12 deficiency (5/18) or iron and folate deficiencies (1/18)) (Figure 4).

## 4. Discussion

Despite the high prevalence of anaemia in different groups studied in Tanzania, data on the elderly population are scarce. Our study provides baseline data in a population of elderly hospitalized patients. The high prevalence (79.5%) of anaemia in the hospitalized elderly revealed in this study is similar to a study done in India in patients attending a geriatric clinic which reported the prevalence of anaemia in elderly to be 71% [26]. These findings are consistent with studies in both developed and developing countries which reveal a higher prevalence of anaemia in hospitalized elderly [27], in contrast to studies done in community elderly where the prevalence ranges between 10.6% and 23% [9, 12, 15, 16, 19, 28]. The higher prevalence in hospital-based studies is not surprising as it is well known that anaemia is a common finding in most disease states particularly if the condition is serious enough to require hospital admission. Developing countries that are still battling the high burden of communicable disease and the concomitant lower socioeconomic status present in these countries are expected to see more hospital admissions of the elderly and are likely to have a higher prevalence of anaemia in the elderly.

More than two thirds of participants in the present study presented with either moderate or severe anaemia. This is in contrary to findings observed from studies done in both community elderly and those in institutions (old age homes), where mild anaemia was the most common type of anaemia [18, 29, 30]. This may be attributed to the hospital-based nature of our study; our population already had relatively progressed illnesses that required admission. However, it may also be possible that there is already a significant proportion of the elderly population in our community that has anaemia in its milder forms. When they acquire conditions that force them to seek medical care, the anaemia would have worsened and thus present with a moderate anaemia or severe anaemia. It is important to also note that the diagnostic criteria used to define anaemia in the elderly in the present study is based on the WHO criterion which was extrapolated from epidemiologic data collected from those under the age of 65 years. It has been argued that these criteria may not be appropriate for the elderly population [5]. Studies conducted on healthy elderly individuals showed a decline in hemoglobin and red cell counts with increasing age in males [6]; it is therefore possible that what we are considering as anaemia in the elderly may in actual sense be the norm in this population. Studies performed on a larger data-base' with participants over 60 years of age are needed to develop a clearly defined diagnostic criteria of anaemia in the elderly. Community-based studies to determine the prevalence and severity of anaemia in healthy Tanzanian elderly population would also add value to what has been observed from this hospital-based study.

In our study, almost a quarter of all participants with anaemia were found to have a nutritional deficiency. Studies in developed countries have reported nutritional deficiency to be the most common cause of anaemia in the elderly (one third of all cases of anaemia) [23]. Although our study did not ascertain other causes of anaemia, we would expect similar findings in a country where nutritional deficiency plays a prime causal role in the development of anaemia in other population groups such as under-fives, adolescent girls, and pregnant women [20, 21, 31]. However, in the present study, the most frequent type of nutritional deficiency was combined deficiency anaemia (iron deficiency and vitamin B12 deficiency or iron deficiency and folate deficiency). Frequency was similar in isolated iron deficiency and isolated folate deficiency. This is contrary to findings from other studies where iron deficiency has dominated the picture in nutritional deficiency anaemia [32, 33]. In the developing world, however, the frequencies are highly variable. For instance, a study in India reported vitamin B12 deficiency as the most frequent cause of nutritional deficiency anaemia [15], whilst in Zimbabwe, folate deficiency was reported as the most frequent cause [34] and a study in Uganda reported iron deficiency as the most frequent cause of nutritional deficiency anaemia [19]. It is however important to interpret these findings in light of the fact that the cutoff levels for diagnosis of IDA in a geriatric hospitalized population in regions with high prevalence of infectious diseases have not been clearly established, an observation that was also previously made in a study by Mugisha et al. [19]. Furthermore, in the present study, only serum ferritin was used as an indicator of iron deficiency anaemia. Serum ferritin is an acute phase reactant whose levels have been shown to increase with age and may be elevated in inflammatory conditions [35, 36]. Nevertheless, different socio-ultural practices (veganism, alcoholism, etc) may play roles in the variations observed, and further research is necessary to establish causal factors. A follow-up epidemiological study on causes of anaemia in the elderly in Tanzania would be of great value.

## 5. Conclusion

The prevalence of anaemia in the hospitalized elderly population in Tanzania is very high and mostly present in moderate and severe forms. Nutritional deficiency anaemia is common, accounting for a quarter of the diagnosed anaemia in the hospitalized elderly. Combined deficiency anaemia (either iron and vitamin B12 deficiency or iron and folate deficiency) is the leading subtype of micronutrient deficiency anaemia. Larger community-based studies are required to define criterion for the diagnosis of anaemia in older individuals and to establish the magnitude of anaemia in the elderly population. Furthermore, the high prevalence calls for a follow up study on the aetiological profile of anaemia in this population.

## Figures and Tables

**Table 1:** Description of study participants.

	All (*N* (%))	Males (*N* = 95) (*N* (%))	Females (*N* = 61) (*N* (%))
*Age* (*years*)			
60–69	98 (62.8)	55 (56.1)	43 (43.9)
70–79	42 (26.9)	29 (69.0)	13 (31.0)
80+	16 (10.3)	11(69.8)	5 (31.2)

*Marital status*			
Married	110 (70.5)	76 (69.1)	34 (30.9)
Unmarried	46 (29.5)	19 (41.3)	27 (58.7)

*Education level*			
None/informal	24 (15.4)	9 (37.5)	15 (62.5)
Primary	54 (34.6)	35 (64.8)	19 (35.2)
Secondary	62 (39.7)	40 (64.5)	22 (35.5)
Higher learning	16 (10.3)	11 (68.75)	5 (31.25)

*BMI* (*kg/m*^*2*^)			
Underweight	4 (2.6)	2 (50.0)	2 (50.0)
Normal	82 (52.6)	43 (52.4)	39 (47.6)
Overweight	64 (41.0)	45 (70.3)	19 (29.7)
Obese	6 (3.8)	5 (83.3)	1 (16.7)

*Blood Pressure*			
Normal	82 (52.6)	52 (63.4)	30 (36.6)
Prehypertensive	42 (26.9)	21 (50.0)	21 (50.0)
Stage I hypertension	27 (17.3)	19 (70.4)	8 (29.6)
Stage II hypertension	5 (3.2)	3 (60.0)	2 (40.0)

## Data Availability

The data supporting the findings of this study are available from the corresponding author upon reasonable request.
